# Low-Cost Honeycomb Biomass Adsorbent for Efficient Pt Recovery from Automobile Catalyst Waste

**DOI:** 10.3390/molecules30142910

**Published:** 2025-07-10

**Authors:** Rafał Olchowski, Patryk Szymczak, Ryszard Dobrowolski

**Affiliations:** 1 Department of Pharmacology, Toxicology and Environmental Protection, Faculty of Veterinary Medicine, University of Life Sciences, Akademicka Sq. 12, 20-950 Lublin, Poland; rafal.olchowski@up.lublin.pl; 2 Department of Analytical Chemistry, Faculty of Chemistry, Institute of Chemical Sciences, Maria Curie-Sklodowska University, M. C. Sklodowska Sq. 3, 20-031 Lublin, Poland; patrick.szymczak18@gmail.com

**Keywords:** biomaterial, Soxhlet extraction, Pt(IV) adsorption, adsorption mechanism

## Abstract

Spent automobile catalysts can be an important source of platinum for industry applications. Low-cost and simple technologies for platinum recovery from this source are sought, especially involving the application of green adsorbents. Honeycomb biowaste can be an excellent candidate for this purpose; n-hexane-treated honeycomb biowaste is therefore obtained for the first time. This material is characterized using several instrumental techniques, confirming the presence of O, N, and P heteroatoms on its surface and the complex morphology of its particles. The maximum static Pt(II)/Pt(IV) adsorption (46 mg/g and 60 mg/g, respectively) onto the n-hexane-extracted honeycomb biomass is reached at pH = 1.55 and a contact time of 50 h. The adsorption kinetics are best fitted to the pseudo-second-order model in both cases. The Langmuir model best described the Pt(II)/Pt(IV) adsorption isotherms on the studied material. Quantitative desorption of the Pt from the studied material is reached for 1 mol/L thiourea dissolved in HCl. The adsorption mechanism of Pt(IV) ions onto the obtained material is based mainly on the surface complexation reactions. The studied material is successfully applied for the first time for Pt(IV) removal from a spent automobile catalyst leachate.

## 1. Introduction

Automobile engines emit harmful exhaust gases (e.g., CO_2_, CH_4_, N_2_O) to the environment [1,2]. The average family car without a catalytic converter can emit ca. 1.5 Mg of toxic gases/year [3]. In order to reduce emissions, automobile catalytic converters (ACCs) are mounted on each produced vehicle. ACCs convert the exhaust gases into more environmentally friendly compounds. There are many types of ACCs, of which three-way catalytic converters (TWCCs) are the most frequently used. They possess a honeycomb cordierite structure coated with γ-Al_2_O_3_ catalyst support. On the surface of the alumina, platinum group metal (PGM) catalysts (0.10–0.15%) are present. The molar ratios of the PGM catalysts used for TWCCs are 5:1 for Pt/Rh and 7:1 for Pd/Rh. Pt is the most common PGM in TWCCs (53%). The worldwide demand for PGMs is continuously increasing (over 200 Mg/year for Pt and Pd) and natural sources of PGMs are strictly limited. Thus, PGMs are very expensive. Moreover, the huge amount of spent automobile catalysts (SACs) can be harmful for the environment due to the toxicity of PGMs to living organisms [4,5]. To avoid these problems, recovery strategies for PGMs from SACs are applied and can be pyrometallurgical, biometallurgical, or hydrometallurgical. In pyrometallurgical methods, crushed carriers containing PGMs are melted with the addition of the other metal. PGMs pass into the metal alloy, while the alumina carrier is separated and scrapped. Biometallurgical processing is based on the application of microorganisms to separate and enrich the PGMs from the Al_2_O_3_ matrix. Meanwhile, in the hydrometallurgical method, the PGMs are leached to the liquid medium (e.g., HCl with oxidants, cyanide, halide, or thiosystems) as chloro-complex compounds. This method requires the application of a separation and refining system (precipitation, electrolysis, extraction, and adsorption). The adsorption is an economic, easy, and eco-friendly process in which PGMs can be selectively and effectively separated and enriched. Additionally, the adsorption of PGMs from SACs onto a solid matrix can provide new interesting catalysts [6,7,8,9,10]. Many porous solids with a large specific surface area and various functionalities can be used during the adsorption process of PGMs (e.g., activated carbon [11], mesoporous organosilica [12], biochar [13], chitosan [14], molecular-ion-imprinted polymer [15]). From the wide range of adsorbents which could possibly be applied in this field, honeycomb biomass deserves special attention as a green adsorbent [16].

Honeycomb biomass is a global-scale waste produced in quantities of thousands of tons/year and contains larval remains, wax, microbes, heavy metals, pesticides, propolis, and honeybee silk. Around 40 wt. % of the total nest mass is honeybee silk. The silk is produced by honeybee larvae before their pupation. It has a coiled molecular structure, unlike spider and silkworm silks. It is built from small, α-helical proteins (ca. 30 kDa) with an alanine-rich core. The diameter of the honeybee silk is in the range of 3.9–6.9 µm. It provides thermal and mechanical stability for the hive. Honeybee silk is a rich source of oxygen and nitrogen heteroatoms, which can be active centers for PGM adsorption. The application of honeycomb biomass for PGM adsorption can prevent environmental pollution from this biowaste and reduce costs of the PGM refining process [17,18,19,20,21,22].

In this work, honeybee biomass extracted with n-hexane is studied for the first time for Pt(II) and Pt(IV) removal from automobile catalyst leachate. The studied material is characterized by various physicochemical techniques. Pt(II) and Pt(IV) adsorption onto the studied material is optimized with some factors (Pt(II)/Pt(IV) solution pH, contact time, Pt concentration). The Pt(II)/Pt(IV) adsorption mechanism onto the studied biosorbent is also studied in detail. Furthermore, studies on Pt desorption from Pt-loaded biosorbent are conducted with HCl and thiourea aqueous solutions. Finally, the newly obtained biosorbent is successfully applied for Pt recycling from the model automobile catalyst leachate.

## 2. Results and Discussion

### 2.1. Physicochemical Characteristics of the Studied Biosorbent

**Figure 1:**
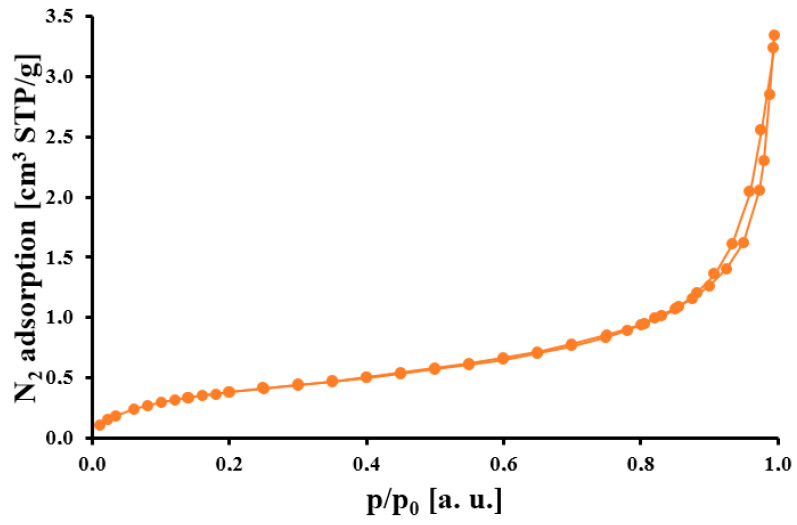
Nitrogen adsorption/desorption isotherm for studied material.

In Table 1, the porosity data for the obtained Extracted Honeycomb Biomass (EHB) material are presented. The studied biomaterial is characterized by an underdeveloped porosity structure with small values of specific surface area (1.5 m^2^/g) and pore volume (0.005 cm^3^/g). The limited pore structure of this material consisted of macropores with an 80 nm diameter. On the contrary, the pristine honeycomb biomass before n-hexane extraction had a specific surface area of 0.8 m^2^/g and a pore volume of 0.002 cm^3^/g. It means that the n-hexane extraction of the pristine material revealed a surface with functional groups, probably by partially removing the waxes and honey from the surface. Additionally, the nitrogen isotherm of the EHB material (Figure 1) is type II with a hysteresis loop at p/p_0_ = 0.9, which confirmed the macroporous structure of the studied material [23].

Results from the elemental composition (CHN, XPS, and EDS analyses) of the studied material revealed the presence of C (47.8–71.9 wt. %), O (21.4–34.3 wt. %), N (5.31–13.3 wt. %), H (8.8 wt. %), and P (1.43 wt. %). Other elements, like K, Al, Mg, Ca, S, Na, Fe, Si, and Cl, are present in this biomaterial below 2 wt. % (Table 1). Honeycomb biomass, which is the basis of the EHB material, contained some organic compounds with both natural (wax, honey, propolis, bee, parasite, and bacteria residues, honeybee silk) and anthropogenic origin (wire residues, pesticides) [17,18,19,20,21]. Thus, the elemental composition of the EHB material is rich. Moreover, a significant variation of the element content is observed (higher C, O, and N content on the surface of the studied material than in its bulk). It could be related to the heterogenous distribution of the C, O, and N in the studied material. The presence of the heteroatoms (O, N, and P) in the EHB material could be useful as active centers for adsorption of heavy metals (like Pt).

The chemical nature of the EHB material surface could be studied by the pH measurement of the distilled water with the immersed EHB material (pH_EHB_). The obtained value (5.8) means that the surface of the EHB material is mainly acidic, with acidic organic functional groups, such as carboxyl, phosphoric acid, and hydroxyl groups [22]. These can dissociate in the water and lower its pH, according to the following equations:

Surf.-COOH + H_2_O ←→ Surf.-COO^−^ + H_3_O^+^ (3 ≤ pKa ≤ 6) (1)

Surf.-OH + H_2_O ←→ Surf.-O^−^ + H_3_O^+^ (8 ≤ pKa ≤ 11) (2)

Surf.-P-OH + H_2_O ←→ Surf.-P-O^−^ + H_3_O^+^ (2 ≤ pKa ≤ 12) (3)

**Figure 2:**
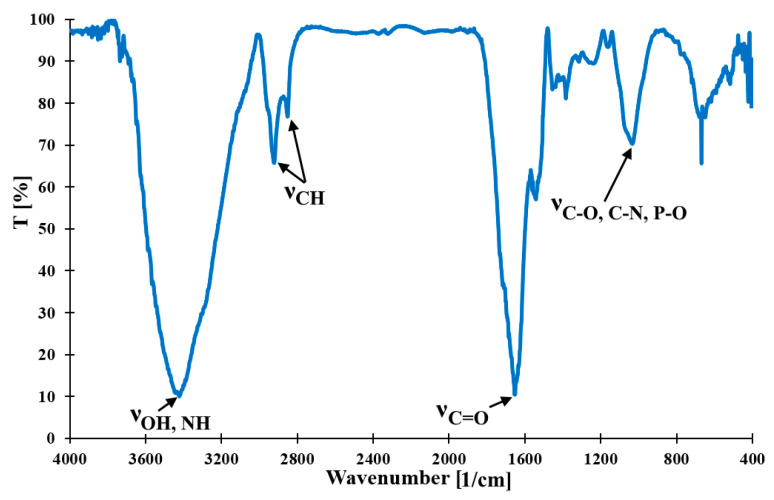
FT-IR spectrum of the studied material.

In Figure 2 the FT-IR spectrum of the studied EHB material is presented. The following spectral bands and corresponding functional groups are recorded: ν_OH, NH_ (3420 1/cm), ν_CH_ (2920 1/cm, 2850 1/cm), ν_C=O_ (1650 1/cm), and ν_C-O, C-N, P-O_ (1030 1/cm) [24]. Based on these results it can be stated that the surface of the EHB material probably consisted of hydroxyl, amine, amide, carboxyl, carbonyl, ester, ether, and phosphoric acid groups. The high-resolution XPS spectra (C 1s (285.0 eV), O 1s (532.5 eV), and N 1s (399.8 eV)) for the studied material are recorded to confirm these guesses. From the deconvolution of the C 1s spectrum four signals are obtained: C-Csp^3^, C-H (285.0 eV; 66.3%), C-OH, C-O-C, C-N (286.5 eV; 23.0%), C=O (288.1 eV; 7.7%), and COOR (289.1 eV; 3.0%). In the case of O 1s deconvolution two signals are revealed: C=O, O=C-O- (531.8 eV; 45.9%) and C-OH, O=C-O- (533.1 eV; 54.1%). Finally, the N 1s spectrum consisted of one signal, -NH- (400.5 eV; 100%) [25,26,27]. In the parentheses the binding energy and the signal intensity contribution are presented. The XPS data for EHB material provide confirmation of the presence of these surface functionalities. The relatively significant amount of the aliphatic carbon and C-O/C-N groups could be related to the hydrocarbons that originated from propolis, residues of waxes, and honeybee silk [17,18,19,20,21]. Thanks to the wealth of surface functionalities of the EHB material it could be successfully applied as an efficient adsorbent of environmental pollutants, both organic and inorganic.

**Figure 3:**
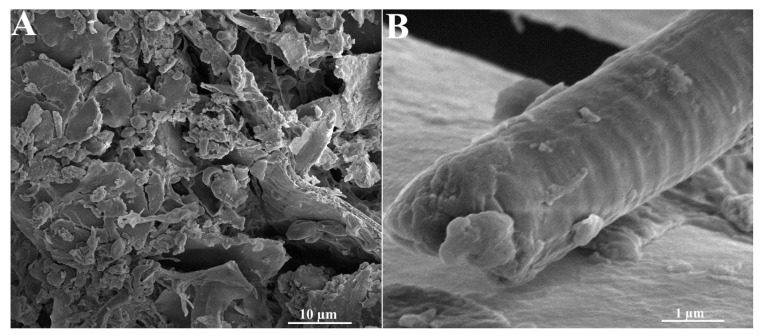
SEM microphotographs of EHB material, (**A**) magnif. 2.5 kx, (**B**) magnif. 25 kx.

In Figure 3 the SEM microphotographs of the EHB material are presented. In the microphotographs the residues of bioorganic matter (e.g., larvae, parasites), crystals of inorganic compounds (Figure 3A), and probably honeybee silk (Figure 3B) can be seen. The diameter of the honeybee silk fiber observed in Figure 3B could be ca. 2.5 µm, which is slightly smaller than that described in the literature [17,18,19,20,21]. Moreover, the coil-like structure could be also observed. It means that the morphology of the EHB particles is complex.

### 2.2. Pt(II) and Pt(IV) Adsorption Studies

**Figure 4:**
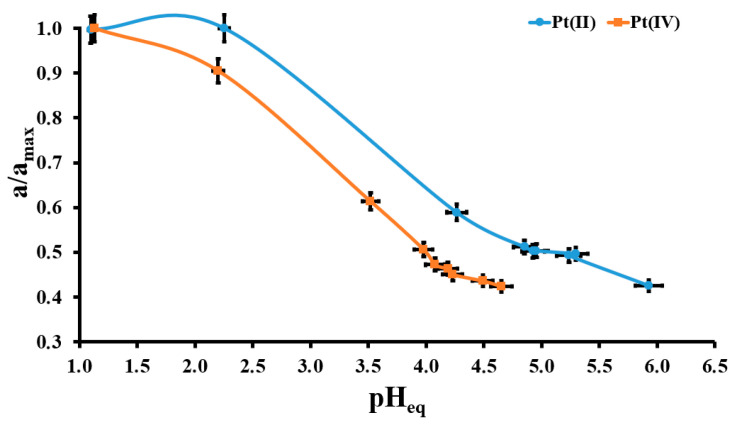
The pH effect for Pt(IV) and Pt(II) adsorption onto the EHB material (m = 20 mg, V = 5 mL, C_0Pt(II)_ = 133 mg/L, C_0Pt(IV)_ = 85 mg/L, a_max_Pt(II)_ = 26.7 mg/g, a_max_Pt(IV)_ = 18.9 mg/g, t_ads._ = 72 h); error bars denote standard deviations from 3 replicates.

The pH effect on the Pt(II) and Pt(IV) adsorption onto the EHB material is presented in Figure 4. In the case of the Pt(II) ions the highest adsorption onto the studied material is observed in the pH_eq_ range of 1.00–2.25. An increase in the pH_eq_ resulted in less adsorption of these Pt species onto the EHB sample, up to ca. 40% of the highest adsorption value. A similar trend is observed for the Pt(IV) ions: the highest adsorption is seen at pH_eq_ = 1.10 and 40% of the highest adsorption value is observed at pH_eq_ = 4.60. At solution pH < 2.0 for Pt(II) and <1.5 for Pt(IV) these Pt species are present in their negative charged forms (PtCl_4_^2−^, PtCl_6_^2−^) [12,28]. At the same time, the surface of the EHB material is positively charged (pH < pH_EHB_), which means that both Pt species could be easily electrostatically attracted by the EHB surface and it is the reason for the highest Pt(II) and Pt(IV) adsorption values in these conditions. The increase in the solution pH resulted in the formation of the hydroxy- and aquacomplexes of Pt(II) and Pt(IV) with slightly negative (PtCl_3_(H_2_O)^−^, PtCl_5_(H_2_O)^−^) or neutral charge (PtCl_2_(H_2_O)_2_, PtCl_3_(OH)_2_(H_2_O)) [12,28] and simultaneously the increasing lead of the negative charge of the EHB surface. These two factors influenced the decrease in the values of the Pt(II) and Pt(IV) adsorption onto the studied adsorbent. For further studies on Pt(II) and Pt(IV) adsorption onto the EHB material, pH_eq_ = 1.55 is chosen.

**Figure 5:**
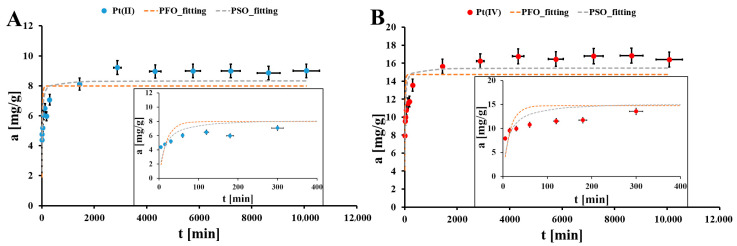
The Pt(II) (**A**) and Pt(IV) (**B**) adsorption kinetics onto the EHB material (m = 20 mg, V = 5 mL, pH_eq_ = 1.55, C_0Pt(II)_ = 39.9 mg/L, C_0Pt(IV)_ = 73.4 mg/L); error bars denote standard deviations from 3 replicates.

Also, the contact time between the Pt(II) and Pt(IV) solution and the EHB material is optimized (Figure 5). In both cases the equilibrium state is reached after 3000 min. There were two stages of the process of Pt(II) and Pt(IV) adsorption onto the studied material. The first stage is fast (up to 300 min) and the second is slow. The first stage could be connected to the Pt(II)/Pt(IV) fast mass transfer from the solution bulk to the EHB surface. In turn, the second step could be related to the slow complexation surface reaction between Pt(II)/Pt(IV) ions and the EHB surface functionalities, which could be slowed by the sterical hindrance during formation of surface complexes [29].

In Table 2 the results of the Pt(II)/Pt(IV) adsorption kinetics data fitting to two theoretical kinetics models, pseudo-first-order (4) and pseudo-second-order (5), are presented. These models are described by the following nonlinear equations [30]:

(4) $$a_{t}=a_{eq}\left(1-e^{-k_{PFO}t}\right)$$

(5) $$a_{t}=\frac{a_{eq}^{2}k_{PSO}t}{1+a_{eq}k_{PSO}t}$$

where a_eq_ is the Pt(II)/Pt(IV) adsorption onto the studied material under equilibrium conditions [mg/g], a_t_ is the Pt(II)/Pt(IV) adsorption onto the EHB material after time t [mg/g], k_PFO_ is the kinetics rate constant for the pseudo-first-order model [1/min], t is the contact time between the Pt(II)/Pt(IV) aqueous solution and the EHB particles [min], and k_PSO_ is the kinetics rate constant for the pseudo-second-order model [g/(mg min)] [30]. The best fitting of the Pt(II) and Pt(IV) adsorption kinetics onto the EHB material is estimated for the PSO model (R^2^ = 0.677–0.691 and similar values for a_eq,theor._ and a_eq, exp._). It meant that the critical factor for adsorption kinetics in the studied system is the surface complexation reaction between Pt(II)/Pt(IV) ions and the EHB surface functional groups [12].

**Figure 6:**
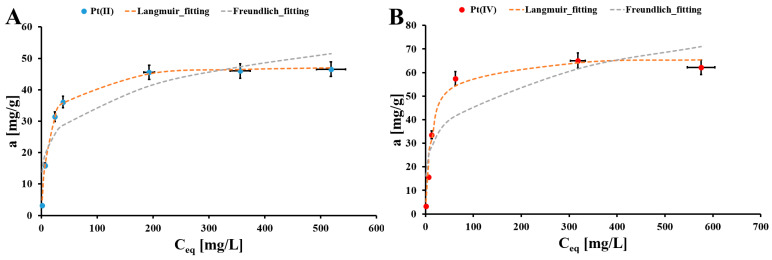
Adsorption isotherms for Pt(II) (**A**) and Pt(IV) (**B**) onto the EHB material (m = 20 mg, V = 5 mL, t_eq_ = 72 h, pH_eq_ = 1.55, a_max_Pt(II)_ = 46 mg/g, a_max_Pt(IV)_ = 60 mg/g, T = (20 ± 4) °C); error bars denote standard deviations from 3 replicates.

In Figure 6 the Pt(II) and Pt(IV) adsorption isotherms for the EHB material are presented. The highest static adsorption capacities for Pt(II) and Pt(IV) in the studied adsorption systems are estimated as 46 mg/g and 60 mg/g, respectively.

The obtained adsorption isotherms in the studied adsorption systems are fitted to two theoretical models: Langmuir (6) and Freundlich (7) (Table 3), which are described by the following nonlinear formulas [31]:

(6) $$a_{eq}=\frac{a_{m}C_{eq}k_{L}}{1+C_{eq}k_{L}}$$

(7) $$a_{eq}=k_{F}C_{eq}^{n_{F}}$$

where a_m_ is the maximum static adsorption capacity of the Pt(II)/Pt(IV) onto the EHB material [mg/g], C_eq_ is the equilibrium concentration of the Pt(II)/Pt(IV) ions [mg/L], k_L_ is the Langmuir equilibrium constant [L/mg], k_F_ is the Freundlich equilibrium constant [mg^1−nF^L^nF^/g], and n_F_ is the Freundlich constant [31]. The Langmuir model is the best fitted to the Pt(II)/Pt(IV) adsorption isotherm experimental data (R^2^ = 0.986–0.998 and similar values of a_m_ and a_max_). It suggested the monolayer chemisorption of the Pt(II) and Pt(IV) ions onto the EHB surface. Moreover, estimated n_F_ values for both Pt(II) and Pt(IV) ions are between 0 and 1, which could be the result of the energetically heterogenous surface of the EHB material [31].

Comparing the Pt(IV) adsorption properties of our adsorbent with other materials, it can be stated that the ion-imprinted ordered mesoporous silica provided higher adsorption capacity (175 mg/g) for Pt(IV) ions after a longer equilibration time (120 h) [32]. On the contrary, the spent-brewer’s-yeast-functionalized zeolite is characterized by a lower Pt(IV) adsorption capacity (0.41 mg/g) for a shorter equilibration time (5 h) than our n-hexane-extracted honeycomb biomass [33].

### 2.3. Pt(IV) Adsorption Mechanism Study

Due to the fact that Pt(II) can be easily oxidized in an aqueous solution [34], our studies concerned the mechanism of Pt(IV) adsorption onto the EHB material. According to XPS data (Table S1) for the pristine EHB material and Pt-loaded EHB it turned out that the C and O content decreased by 4.6 wt. % and 2.6 wt. %, respectively. Moreover, the N content change is negligible and Cl with Pt appeared on the studied material surface (Cl: 2.77 wt. % and Pt: 5.39 wt. %). Probably, Pt(IV) ions adsorbed on the EHB surface as chloride complexes and catalyzed the redox process with the surface functionalities. This hypothesis can be partially based on the high-resolution XPS data (Table S1). Pt(IV) ions did not change oxidation level during the adsorption process on the EHB surface. In turn, the C content in the surface functionalities of the EHB material (C-OH, C-O-C, C-N, C=O, and COOR) did not change substantially (by 0.7–0.8 wt. %) and the O content in these groups changed noticeably by 11.5 wt. % (increase in the C-OH, C-O-C, and O=C-O- groups and decrease in the C=O and O=C-O- groups). It suggested that the Pt(IV) adsorption process on the EHB material could be complex, regarding the redox reactions on the EHB surface, the electrostatic attraction of the Pt(IV) chloride complexes, and the surface complexation. The surface complexation could be the main mechanism. This chemisorption is also confirmed by the second slow stage in the kinetics studies and by the best Langmuir model fitting to the isotherm data.

### 2.4. Desorption Study

**Figure 7:**
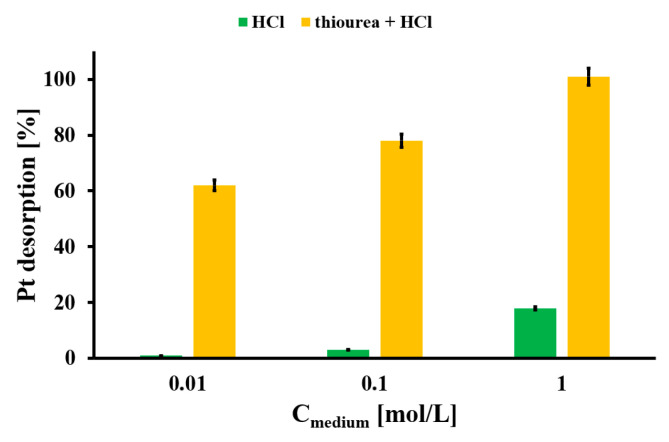
Pt desorption from Pt-loaded EHB material with varying liquid media (m_des._ = 20 mg, V_medium_ = 5 mL, t_des._ = 24 h, A_Pt_ = 26.6 mg/g); error bars denote standard deviations from 3 replicates.

A study of the desorption of the Pt from Pt-loaded EHB material was carried out. The efficient regeneration of the used adsorbent could provide some benefits, like low-cost Pt removal related to the reuse of the EHB material from automobile catalyst leachate and environmental care. In Figure 7 the results of Pt desorption from the Pt-loaded EHB material by two varying liquid media (HCl and thiourea dissolved in HCl) are presented. The quantitative desorption of the Pt from the Pt-loaded EHB is obtained for 1 mol/L thiourea dissolved in HCl. The HCl by itself provided negligible Pt desorption from the studied biomaterial. The best results using thiourea HCl solution could be the result of the presence of the -S and -NH_2_ functionalities in the thiourea molecules. These functionalities could form a sustainable complex with Pt, stronger than that presented on the Pt-loaded EHB surface [35]. The EHB surface could be slightly changed after thiourea desorption of Pt, so the second surface reaction between Pt(IV) ions and the altered EHB surface could occur but to a lesser extent.

### 2.5. Pt(IV) Removal from Automobile Catalyst Leachate

**Figure 8:**
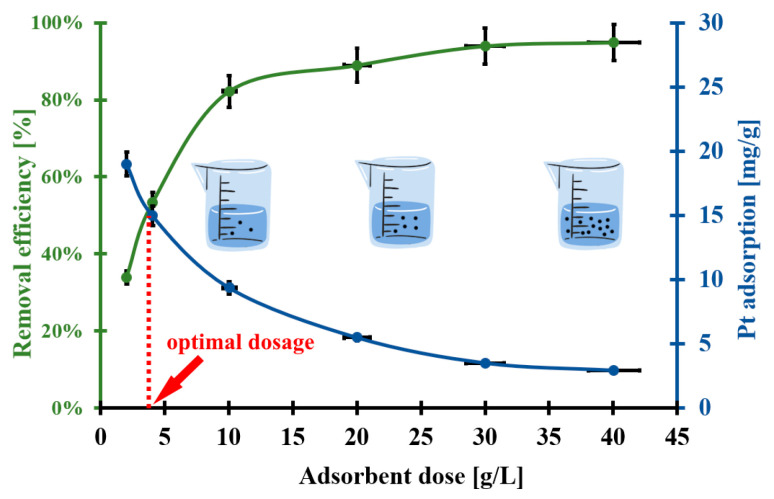
The effect of EHB biosorbent dosage on (a) Pt removal efficiency and (b) Pt adsorption capacity from the matrix of an unused automotive catalyst in an adsorption system: (pH_0_ = 1.5, C_0Pt_ = 188 mg/L, t = 48 h, V_med._= 5 mL, T = (20 ± 4) °C); error bars denote standard deviations from 3 replicates.

The EHB material is applied for Pt(IV) ion removal from the model automobile catalyst leachate solution. The Pt removal efficiency and adsorption of Pt vs. EHB biomaterial dosage are presented in Figure 8. It can be seen that higher doses of the studied adsorbent resulted in a decrease in the Pt adsorption (from 20 mg/g to 3 mg/g). Simultaneously, the Pt removal efficiency rose from 30% to 100% and then a plateau is observed. These trends could be the result of the gradual saturation of active sites present on the EHB surface—firstly the primary active sites and secondly the other active sites—and the drop in the effective surface area [13]. The optimal dosage of the biosorbent is 4 g/L, which corresponded to both the highest Pt(IV) removal efficiency and Pt adsorption. The application of this relatively low optimal dosage of the biosorbent for Pt(IV) removal from the studied leachate could result in lower costs, a more eco-friendly approach, and ease of industrial-scale transfer.

## 3. Materials and Methods

### 3.1. Materials and Reagents

The following reagents are used: sodium hydroxide (>95%), hydrochloric acid (ca. 36%, Suprapur), n-hexane (>99%), hydrogen peroxide (30%), potassium tetrachloroplatinate(II) (>99%), potassium hexachloroplatinate(IV) (>99%), thiourea (>99%), and standard Pt solution (1000 mg L^−1^), all obtained from Merck (Darmstadt, Germany).

European Reference Material ERM^®^-EB503a (LGC Standards, Łomianki, Poland) (unused powdered automobile catalyst) from BAM is used. Double-distilled Milli-Q water from Millipore (Merck, Darmstadt, Germany) is also used.

The “Miodek” Apiary Farm in Lublin (Poland) is the manufacturer of the starting material (pristine honeycomb biomass (PHB)) [36].

### 3.2. Biosorbent Preparation

The pristine honeycomb biomass is dried in a laboratory oven at 120 °C for 24 h. The dried material is ground in a porcelain mortar with liquid nitrogen. The ground biomass is purified of metal residue with a strong magnet. Next, 42 g of this material is placed in a cellulose thimble, which is put inside a Soxhlet apparatus. Then, 250 mL of n-hexane is added to a rounded flask. The Soxhlet extraction of the honeycomb biomass is carried out near the boiling point of n-hexane. It is ended when the solvent present in the deflegmator is clear. After extraction the material is dried in air overnight to remove the n-hexane residues. The obtained material is denoted as EHB.

### 3.3. Instrumentation

The ASAP 2420 (Micromeritics Inc., Norcross, GA, USA) is used for the porosity characterization of the studied honeycomb biomass. Measurements are conducted at −196 °C after the degassing of the studied samples at 120 °C in a vacuum for 12 h. The BET surface area (S_BET_), total pore volume (V_T_), and BJH pore diameter (d_BJH_) are estimated based on the desorption branch of the nitrogen adsorption/desorption isotherm.

A scanning electron microscope (SEM) (Carl Zeiss Ultra Plus, Carl Zeiss, Jena, Germany) equipped with a BrukerAXS energy dispersive X-ray (EDX) detector (Bruker, Karlsruhe, Germany) is applied for the morphological and elemental analysis of the studied material. The SEM is also equipped with secondary electron (SE) and backscattered electron (BSE) detectors. All the experiments are conducted under a 20 kV acceleration voltage and 5 nA probe current.

The Fourier-transform infrared (FT-IR) spectrum of the studied material is recorded by the FT-IR Nicolet 8700A spectrometer (Thermo Scientific, Waltham, MA, USA) in the wavenumber range of 400–4000 1/cm with KBr pellets. X-ray photoelectron spectra (XPS) are recorded by using a Multi-Chamber Analytical System (Prevac, Rogów, Poland) equipped with monochromatic Al K_α_ radiation (1486.6 eV) (Gammadata Scienta, Uppsala, Sweden) and an X-ray power of 450 W. The carbon C1s peak at 285 eV is a reference for all binding energies. Recorded FT-IR and XPS spectra are used for identification of the surface functionalities on the studied material.

The CHN elemental analyses are performed with the EA 3000 Elemental Analyzer (Euro Vector, Italy). CHN and XPS data are applied for the study of the elemental structure of the studied material.

The pH measurements are performed by using the pH meter CP-401 (Elmetron, Zabrze, Poland) equipped with a glass electrode after suitable calibration.

The Pt in an aqueous phase is determined with the electrothermal atomic absorption spectrometer (ET AAS) SpectrAA 880Z (Varian, Belrose, Australia) equipped with a deuterium background correction system and Pt hollow cathode lamp (Varian, Australia). The measurement parameters are presented in Table S2.

### 3.4. Pt Adsorption Studies

The static adsorption studies of two Pt species (Pt(II) and Pt(IV)) are performed at (20 ± 4) °C. First, 20 mg of the studied material is mixed with 5 mL of an aqueous Pt(II) or Pt(IV) solution with initial pH adjusted to 1.55, except for in pH effect studies, where the initial solution pH is in the range of 1–9. The obtained suspension is shaken at 115 rpm for 72 h, except for in the adsorption kinetics studies, where the contact time between the solid and liquid phases is in the range of 5–10,000 min. The Pt(II) and Pt(IV) adsorption isotherms are estimated for the initial Pt(II) and Pt(IV) concentrations between 14.0 mg/L and 822 mg/L. After a defined contact time, the studied adsorbent is separated from the aqueous solution by means of centrifugation. The initial and final Pt concentrations are determined in the liquid phase by the ET AAS technique.

The adsorbed amount of Pt(II) or Pt(IV) (in [mg/g]) onto the surface of the studied material is calculated according to the equation below (Equation (8)):

(8) $$a=\frac{\left(C_{0}-C_{eq}\right)V}{m}$$

where C_0_ is the initial Pt(II) or Pt(IV) concentration in an aqueous solution [mg/L], C_eq_ is the final Pt(II) or Pt(IV) concentration in an aqueous solution [mg/L], V is the volume of the aqueous solution containing Pt(II) or Pt(IV) ions [mL], and m is the adsorbent mass [mg].

The studies of the desorption of Pt from the Pt-loaded EHB material are performed in two varying media: HCl and thiourea + HCl with a concentration range 0.01–1.00 mol/L. First, 20 mg of Pt-loaded EHB is shaken with 5 mL of a liquid medium for 48 h. After that, the liquid phase is separated from the solid phase via centrifugation and the Pt concentration is measured in the liquid phase using the ET AAS technique. The Pt desorption [%] is calculated as follows:

(9) $$Pt\;desorption=\frac{C_{Pt\_des.}V_{med.}}{m_{Pt\_loaded\_EHB}A_{Pt}}100\%$$

where C_Pt_des._ is the concentration of the desorbed Pt [mg/L], V_med._ is the volume of the liquid medium [mL], m_Pt_loaded_EHB_ is the mass of the Pt-loaded EHB [mg], and A_Pt_ is the Pt content in the Pt-loaded EHB [mg/g].

### 3.5. Pt Removal from Spent Automobile Catalyst Leachate

Studies regarding the Pt removal from the model spent automobile catalyst leachate by the EHB material are performed. The European Reference Material ERM^®^-EB504a is used as the model SAC. Briefly, PGMs are leached from the model SAC at 20 °C with a 7% H_2_O_2_ and 3.3% HCl solution [11]. Next, the solution is filtered and then a fixed portion of EHB material is added to 5 mL of the extract. After equilibrium is reached, the platinum concentrations are measured using the ET AAS technique. The platinum removal efficiencies [%] (Equation (10)) are calculated as follows:

(10) $$\mathrm{Removal}\mathrm{efficiency}\left[\%\right]=\frac{\left(C_{0}-C_{eq}\right)}{C_{0}}100\%$$

## 4. Conclusions

The n-hexane extraction of the raw honeycomb biomass resulted in novel biomaterial with a macroporous structure, complex morphology of its particles (e.g., bioorganic matter, inorganic crystals, honeybee silk), and an acidic surface with functionalities containing some heteroatoms like O, N, and P. The heteroatoms partly originated from proteins of honeybee silk fibers, whose coil coiled structure is detected in the synthesized biomaterial. These heteroatoms can be the efficient active centers for Pt(II)/Pt(IV) ion adsorption from an aqueous solution.

Pt(II)/Pt(IV) adsorption equilibrium is established after 50 h, which is related to the reaction between the studied ions and the biosorbent surface. The adsorption kinetics in the two studied adsorption systems is well characterized by the pseudo-second-order model. The highest static adsorption capacities of Pt(II) and Pt(IV) onto the studied biosorbent are 46 mg/g and 60 mg/g, respectively. For both studied adsorption systems the Langmuir model is the best fitted to the adsorption isotherm data, which confirmed the chemisorption nature of the adsorption process.

The adsorption mechanism of Pt(IV) ions on the studied biomaterial is complex, regarding the redox reactions, electrostatic attraction, and the surface complexation as the main surface reaction. The best liquid medium for Pt recovery from the Pt-loaded n-hexane-extracted honeycomb biomass is 1 mol/L thiourea dissolved in HCl (100% recovery).

The n-hexane-extracted honeycomb biomass is successfully applied for Pt(IV) removal from the spent automobile catalyst leachate with an optimal dose of 4 g/L. The low dosage of the material can decrease costs of its usage and it allows easy industrial-scale transfer. Moreover, the reuse of huge amounts of honeybee biomass waste can be an eco-friendly approach. It is a promising technique to recover Pt from spent automobile catalysts, but further studies should be performed regarding the multiple reusage of the studied adsorbent.

## Figures and Tables

**Table 1:** Results from nitrogen adsorption/desorption, elemental analysis (CHN, EDS, XPS), and pH studies for EHB sample.

Porosity Data			CHN			XPS				EDS ^$^			pH_EHB_^&^
S_BET_ [m^2^/g]	V_T_ [cm^3^/g] 10^−3^	d_BJH,des._ [nm]	C [wt. %]	N [wt. %]	H [wt. %]	C [wt. %]	O [wt. %]	N [wt. %]	P [wt. %]	C [wt. %]	O [wt. %]	N [wt. %]	5.8 * ± 0.3 ^#^
1.5 * ± 0.3 ^#^	5 * ± 1 ^#^	80 * ± 16 ^#^	47.8 * ± 0.15 ^#^	6.27 * ± 0.13 ^#^	8.8 * ± 0.1 ^#^	71.9 * ± 3.6 ^#^	21.4 * ± 1.1 ^#^	5.31 * ± 0.27 ^#^	1.43 * ± 0.07 ^#^	51.2 * ± 2.5 ^#^	34.3 * ± 1.7 ^#^	13.3 * ± 0.6 ^#^	

*—mean value from 3 replicates, ^#^—standard deviation from 3 replicates, ^$^—other detected elements (K, P, Al, Mg, Ca, S, Na, Fe, Si, Cl) < 2%, ^&^—pH value of distilled water with the immersed EHB material (m/V = 20 mg/5 mL).

**Table 2:** Parameters of Pt(II) and Pt(IV) adsorption kinetics data fitting to theoretical kinetics models, i.e., pseudo-first-order (PFO) and pseudo-second-order (PSO), for studied EHB material.

Pt Species	PFO			PSO			a_eq,exp._ [mg/g]
	a_eq,theor._ [mg/g]	k_PFO_ [1/min]10^−3^	R^2^	a_eq,theor._ [mg/g]	k_PSO_ [g/(mg min)]10^−3^	R^2^	
Pt(II)	7.98 * ± 0.11 ^#^	55 * ± 2.17 ^#^	0.429	8.34 * ± 0.35 ^#^	9.0 * ± 0.1 ^#^	0.677	8.98 * ± 0.17 ^#^
Pt(IV)	14.8 * ± 1.3 ^#^	65 * ± 2.56 ^#^	0.428	15.5 * ± 1.6 ^#^	5.0 * ± 0.1 ^#^	0.691	16.8 * ± 0.3 ^#^

*—mean value from 3 replicates, ^#^—standard deviation from 3 replicates.

**Table 3:** Parameters of Pt(II) and Pt(IV) adsorption isotherm data fitting to theoretical isotherm models, i.e., Langmuir and Freundlich, for studied EHB material.

Pt Species	Langmuir			Freundlich			a_max_ [mg/g]
	a_m_ [mg/g]	k_L_ [L/mg]	R^2^	n_F_ [a. u.]	k_F_ [mg^1−nF^L^nF^/g]	R^2^	
Pt(II)	48.2 * ± 2.1 ^#^	0.07 * ± 0.01 ^#^	0.998	0.23 * ± 0.01 ^#^	12.6 * ± 0.1 ^#^	0.849	46.0 * ± 2.0 ^#^
Pt(IV)	67.1 * ± 1.6 ^#^	0.07 * ± 0.01 ^#^	0.986	0.24 * ± 0.01 ^#^	15.3 * ± 0.1 ^#^	0.823	60.0 * ± 2.3 ^#^

*—mean value from 3 replicates, ^#^—standard deviation from 3 replicates.

## Data Availability

The raw/processed data required to reproduce these findings cannot be shared at this time due to technical or time limitations. Requests to access the datasets should be directed to the corresponding author.

## Supplementary Materials

The following supporting information can be downloaded at: https://www.mdpi.com/article/10.3390/molecules30142910/s1, Table S1: Pt measurement parameters via ET AAS technique; Table S2: XPS data for EHB material before and after Pt(IV) adsorption.
