# Supplementary material for: Low-Cost Honeycomb Biomass Adsorbent for Efficient Pt Recovery from Automobile Catalyst Waste
Source: Molecules. 2025 Jul 10;30(14):2910. doi: 10.3390/molecules30142910 (PMC12300734; doi:10.3390/molecules30142910)
Supplement: Supplementary file 1 [file molecules-30-02910-s001.zip › molecules-3700074-supplementary.pdf]

Low-Cost Honeycomb Biomass Adsorbent for Efficient Pt Recovery from Automobile Catalyst Waste

Authors: Rafał Olchowski[1], Patryk Szymczak[2] and Ryszard Dobrowolski[2,*]

[1] Department of Pharmacology, Toxicology and Environmental Protection, Faculty of Veterinary Medicine, University of Life Sciences, Akademicka St. 12, 20-950 Lublin, Poland
rafal.olchowski@up.lublin.pl

[2] Department of Analytical Chemistry, Institute of Chemical Sciences, Faculty of Chemistry, Maria Curie-Sklodowska University, M. C. Sklodowska Sq. 3, 20-031 Lublin, Poland
ryszard.dobrowolski@mail.umcs.pl; patrick.szymczak18@gmail.com

[*] **Correspondence:** ryszard.dobrowolski@mail.umcs.pl

Table S1. XPS data for EHB material before and after Pt(IV) adsorption.

**Bonding energy [eV]**	**Functional group /energy core level**	**Content [%]**	
		**Before Pt(IV) adsorption**	**After Pt(IV) adsorption**
C1s			
285.0	C-C/C-H	66.3	66.2
286.5	C-OH/C-O-C/C-N	23.0	22.3
288.1	C=O	7.7	8.5
289.1	COOR	3.1	3.0
O1s			
531.8-531.9	C=O/<u>O</u>=C-O-	45.9	34.4
533.1	C-OH/C-O-C/O=C-<u>O</u>-	54.1	65.6
N1s			
400.2-400.5	-NH-	100	100
Cl2p			
198.3-198.6	$Cl2p_{3/2}$	-	50.7
200.0-200.2	$Cl2p_{1/2}$	-	49.3
Pt4f			
73.29-73.44	$Pt4f_{7/2}$	-	51.2
76.59-76.75	$Pt4f_{5/2}$	-	48.8

Table S2. Pt measurement parameters via ET AAS technique.

**Measurement parameter**	**Value**
Lamp current [mA]	10.0
Wavelength [nm]	265.9
Slit width [nm]	0.5
Sample volume [µL]	15
Atomization temperature [°C]	2700
Pyrolysis temperature [°C]	1000
